# A non-traditional crystal-based compound screening method targeting the ATP binding site of *Plasmodium falciparum* GRP78 for identification of novel nucleoside analogues

**DOI:** 10.3389/fmolb.2022.956095

**Published:** 2022-10-07

**Authors:** Alexander Mrozek, Tetyana Antoshchenko, Yun Chen, Carlos Zepeda-Velázquez, David Smil, Nirbhay Kumar, Hua Lu, Hee-Won Park

**Affiliations:** ^1^ Department of Biochemistry & Molecular Biology, Tulane University School of Medicine, New Orleans, LA, United States; ^2^ Drug Discovery Program, Ontario Institute for Cancer Research, Toronto, ON, Canada; ^3^ Department of Global Health, George Washington University Milken Institute of Public Health, Washington, D.C., DC, United States

**Keywords:** *Plasmodium falciparum*, malaria, GRP78, chaperone, unfolded protein response, nucleoside analogues, drug discovery & development

## Abstract

Drug resistance to front-line malarial treatments represents an ongoing threat to control malaria, a vector borne infectious disease. The malarial parasite, *Plasmodium falciparum* has developed genetic variants, conferring resistance to the current standard therapeutic artemisinin and its derivatives commonly referred to as artemisinin-combination therapies (ACTs). Emergence of multi-drug resistance parasite genotypes is a warning of potential treatment failure, reaffirming the urgent and critical need to find and validate alternate drug targets to prevent the spread of disease. An attractive and novel drug target includes glucose-regulated protein 78 kDa (GRP78, or BiP), an essential molecular chaperone protein involved in the unfolded protein response that is upregulated in ACT treated *P. falciparum* parasites. We have shown that both sequence and structure are closely related to human GRP78 (hGRP78), a chaperone belonging to the HSP70 class of ATPase proteins, which is often upregulated in cellular stress responses and cancer. By screening a library of nucleoside analogues, we identified eight ‘hit’ compounds binding at the active site of the ATP binding domain of *P. falciparum* GRP78 using a high-throughput ligand soaking screen using x-ray crystallography. These compounds were further evaluated using protein thermal shift assays to assess target binding activity. The nucleoside analogues identified from our screen provide a starting point for the development of more potent and selective antimalarial inhibitors. In addition, we have established a well-defined, high-throughput crystal-based screening approach that can be applied to many crystallizable *P. falciparum* proteins for generating anti-*Plasmodium* specific compounds.

## Introduction

Malaria is a vector borne infectious disease caused by the protozoan parasite of the *Plasmodium* genus, infecting both the host liver and blood cells causing an estimated 627,000 deaths annually (World Health Organization, 2021). There are five species known to infect humans: *P. falciparum, P. vivax, P. ovalae, P. malariae* and *P. knowelsi*, however *P. falciparum* is by far the most dangerous resulting in the highest associated mortality and morbidity (World Health Organization, 2021). Despite numerous therapeutic and preventative efforts leading to an overall reduction in death and disease over the past decade, the spread of partial resistance to front-line malarial therapeutics such as artemisinin and related artemisinin combination therapies (ACTs) has emerged and continues to spread (Visser et al., 2014; Suresh and Haldar, 2018). Malaria multidrug resistance highlights the critical and urgent need to discover new targets and chemical scaffolds.

One class of druggable targets is the endoplasmic reticulum (ER) resident molecular heat shock chaperone GRP78, which is also referred to as Immunoglobulin Binding Protein (BiP), an HSP70-like stress response protein implicated in the cellular unfolded protein response (UPR). GRP78 is essential in the maintenance of ER homeostasis, where it facilitates proper protein folding, mitigating physiological and pathological damages that may cause an accumulation of misfolded proteins and ER stress (Ibrahim et al., 2019). Moreover, it has been shown that GRP78 from *P. falciparum* (*pf*GRP78) is associated with numerous protein families such as: transcriptional and translational machinery, the proteosome and proteolytic enzymes, as well as proteins involved in physiological and metabolic pathways (Gardner et al., 2013). There is growing evidence *pf*GRP78 is cytoprotective, aiding in adaptation between physiological and environmental changes occurring during transmission from the host vector to the infected cell (Kumar et al., 1988; Przyborski et al., 2015; Day et al., 2019). We hypothesize that up-regulated expression of *pf*GRP78 allows *P*. *falciparum* infected cells to withstand and adapt to host conditions and have improved rates of survival.

The presence of the ER stress-response chaperone *pf*GRP78 in the malaria parasite suggest that this protein could be used as a rational therapeutic target, and various studies including the previous work from our group have explored small molecule inhibitors against *P*. *falciparum* and human HSP70-like chaperones (Frame et al., 2015; Jones et al., 2016; Chen et al., 2018). *pf*GRP78 shares an 84.4% sequence identity to the human orthologue of GRP78 and this value increased to 87.8% when evaluating the nucleotide-binding domain (NBD) only (Chen et al., 2018). GRP78 is structurally divided into two distinct domains: the nucleotide-binding domain at the amino terminus followed by a flexible linker to the substrate-binding domain at the carboxyl-terminus (Chakafana et al., 2019). ATP hydrolysis and ADP/ATP exchange within the NBD of GRP78 play an essential role in the chaperone activity (Pobre et al., 2019). Therefore, the disruption of the interaction of *pf*GRP78-NBD with ATP using nucleoside analogues with varying structural modifications could inhibit *pf*GRP78 functions requiring ATP hydrolysis.

In this present study, we expressed and purified recombinant *pf*GRP78-NBD and the human orthologue hGRP78-NBD from bacteria. We then grew crystals of *pf*GRP78-NBD to perform a novel compound screening assay using adenine-based nucleoside analogues with distinct modifications to interrogate the active site of *pf*GRP78-NBD in effort to identify compounds that bind the *Plasmodium* protein with enhanced selectivity when compared to the human ortholog hGRP78-NBD. Our crystal-based assay identified eight distinct nucleoside analogues present at the active site of *pf*GRP78-NBD in the complex crystal structures. We then used a protein Thermal Shift Assay (TSA) to further characterize and identify potential differences between the malaria and human chaperone ATP binding sites.

## Materials and methods

### Reagents

All reagents, materials and nucleoside analogues were purchased from commercial suppliers and were used as received unless otherwise noted. HP1, HP2, HP3, HP4, HP5, HP13, HP14 were purchased from Carbosynth. HP18 was purchased from Acros Organics. HP19, HP20, HP21, HP22, HP23, HP24, HP25, HP26, HP27 were purchased from TargetMol. HP28, HP31, HP32 and HP33 were purchased from Selleck Chemicals. HP9, HP10, HP11 and HP12 were synthesized as detailed below.

### DNA cloning, protein expression and purification

The plasmids used in this study have been described previously (Chen et al., 2018). Briefly, the gene encoding for *pf*GRP78-NBD (residues 26-404) and hGRP78-NBD (residues 26-407) were amplified from full-length *pf*GRP78 (residues 26-652) and hGRP78 (residues 1-654) by PCR and ligated into a pET28-MHL vector containing an N-terminal His_6_-tag (GenBank accession EF456735) and expressed in *E*. *coli* BL21 (DE3) competent cells (Thermo Scientific). Bacterial starter cultures were grown in LB medium containing 50 μg/ml kanamycin for 16 h at 37°C and then transferred to Terrific Broth containing 50 μg/ml kanamycin. The cells were further grown until the OD_600nm_ reached ∼2.0 and expression was induced with 0.2 mM isopropyl β-D-1-thiogalactopyranoside at 17°C. Eighteen hours post-induction, the cells were harvested by centrifugation at 10,000 rpm for 20 min at 4°C, resuspended in a binding buffer (20 mM TRIS-HCl pH 7.5, 200 mM NaCl, 1 mM β-mercaptoethanol) supplemented with (1 mM PMSF, 1 mM Benzamidine, 0.1% IGEPAL CA-630) and lysed by sonification. Lysates were clarified by centrifugation at 10,000 rpm for 20 min at 4°C and the supernatant was loaded on to 5 ml column of Ni-NTA agarose beads (Thermo Scientific) pre-equilibrated with the binding buffer. The matrix was washed in a stepwise manner using the binding buffer containing increasing concentrations of imidazole (5 mM, 20 mM, 50 mM) and the protein was eluted with the same buffer containing 250 mM imidazole. The eluate was loaded on to a HiLoad 26/600 Superdex 200 pg column (Cytiva) pre-equilibrated with the binding buffer. Target protein-containing fractions were pooled and concentrated using 10 kDa MWCO centrifugal filters (Amicon), flash frozen and stored at −80°C. Expression and purification of *pf*GRP78 was identical to hGRP78. The N-terminal His_6_-tag was cleaved using a 1:50 ratio of TEV protease at 4°C overnight. TEV-cut *pf*GRP78-NBD was mixed with a 10-fold molar excess of 8-bromoadenosine and was concentrated to ∼30 mg/ml for crystallization.

### Crystallization, DMSO-driven crystal-ligand replacement screening, data collection and structure determination

Initial crystallization trials were conducted using the sitting-drop, vapor diffusion method using in-house crystallization screening kits, with each drop containing a solution of the complex of *pf*GRP78-NBD with 8-bromoadenosine and a reservoir solution by high throughput crystallization robot Mosquito Xtal3 (SPT Labtech). Drops were set in 96-well Intelliplates (Art Robbins Instruments). Crystals appeared within a week in the reservoir solution containing 0.7 M (NH_4_)_2_SO_4_, 1.2 M Li_2_SO_4_, 0.1 M NaCitrate, pH 5.6 at a room temperature. These crystals were then used for crystal-ligand replacement screening using DMSO. Crystals were first subjected to a DMSO soaking test by titrating them with various concentrations of DMSO to determine a critical threshold between surviving and dissolving (5% addition of DMSO to the drop volume dissolved crystals completely, but 4% DMSO kept crystals intact without change in morphology). Subsequent experiments added 5% of DMSO and 5 mM of each nucleoside analogue into the crystal-containing drop for DMSO-driven crystal-ligand replacement screening. Survived crystals for eight different nucleoside analogues were cryoprotected using paratone-N and flash-frozen in liquid nitrogen until data collection. These survived crystals come from bound 8-bromoadenosine being replaced by the added nucleoside analog, resulting in stabilization of proteins in the crystal lattice. The diffraction data were collected at the Canadian Light Source 08B1-1 beamline in Saskatoon, Canada, as well as the Advanced Photon Source 19-ID-D and 23-ID-D beamlines at the Argonne National Laboratory in Chicago, Illinois. Data were processed using XDS (Kabsch, 2010) and all structures were solved by molecular replacement using MOLREP (Vagin and Teplyakov, 2010) in the CCP4 crystallographic suite (Winn et al., 2011). Structures were solved using the ADP bound *pf*GRP78-NBD structure excluding ADP (PDB ID: 5UMB) as a search model. After molecular replacement, each model was subjected to multiple rounds of model building and refinement using COOT (Emsley and Cowtan, 2004) and REFMAC (Murshudov et al., 2011), respectively. Coordinate files for all eight *pf*GRP78-NBD structures have been deposited in the Protein Data Bank with accession codes: 8DIQ (HP2), 8DIP (HP3), 8DIS (HP5), 8D1W (HP10), 8DIY (HP19), 8D20 (HP20), 8D22 (HP22) AND 8D24 (HP23). Structural figures were generated using PyMOL Molecular Graphics System (Version 2.5.0 Schrödinger, LLC) as well as LIGPLOT (Laskowski and Swindells, 2011).

### Thermal shift assay

Thermal shift assay measurements were performed on a QuantStudio 6 Flex Real-Time PCR instrument (Applied Biosystems) in 96-well plates (Axygen) sealed with transparent adhesive film (BioRad). *pf*GRP78-NBD and hGRP78-NBD were diluted to a final concentration of 5 µM in DSF assay buffer (25 mM HEPES pH 7.5, 250 mM NaCl, 1 mM β-mercaptoethanol) and nucleoside analogues were added at a final concentration of 0.5 mM (5% DMSO final concentration). Protein-compound samples were aliquoted in four replicates followed by the addition of the fluorescent probe bis-ANS (Invitrogen) diluted to 50 µM in a 20 µL total reaction volume. Temperature was continuously increased from 10°C to 95°C at an increment of 1°C/min. Melting curves were analyzed using the software Thermal Shift Assay—Curve Rapid and Automatic Fitting Tool (Lee et al., 2019). T_m_ was defined as the temperature corresponding to the maximum value of the first derivative of fluorescence. Error bars shown in Figure 4 represent standard error of the mean and were calculated using GraphPad Prism (version 9.3.1) for macOS (GraphPad Software, San Diego, California, United States, www.graphpad.com/).

### Synthesis of nucleoside analogues HP9, HP10, HP11 and HP12

A series of nucleoside analogues (HP9, HP10, HP11, HP12) with modifications at the C8 position of the adenine moiety were synthesized from a previously described method (Tatani et al., 2015) and all characterizations by LRMS and ^1^H NMR were consistent with reported values.

### Preparation of HP9 ((2*R*,3*R*,4*S*,5*R*)-2-(6-amino-8-(benzyl(methyl)amino)-9*H*-purin-9-yl)-5-(hydroxymethyl)tetrahydrofuran-3,4-diol)

HP9 was synthesized according to the synthesis procedure above from a from a mixture of 8-bromoadenosine (100 mg, 0.289 mmol, 1 eq.), *N*-methyl-1-phenylmethanamine (0.867 mmol, 3 eq.), and *N,N-*diisopropylethylamine (1.73 mmol, 0.302 ml, 6 eq.) was heated to 140°C under microwave irradiation for 10 h. The reaction mixture was then concentrated under reduced pressure, and the residue purified by column chromatography on silica gel (Biotage SNAP 10 g column, 0-40% MeOH/EtOAc as the eluent, 28 CV) to afford the desired product in 56% yield (64 mg). LRMS (M + H)^+^: 387.5 Purity: 98% (UV/254 nm).

### Preparation of HP10 ((2R,3R,4S,5R)-2-(6-amino-8-((2-chlorobenzyl)amino)-9H-purin-9-yl)-5-(hydroxymethyl)tetrahydrofuran-3,4-diol)

HP10 was synthesized according to the synthesis procedure above from a mixture of 8-bromoadenosine (100 mg, 0.289 mmol, 1 eq.), (2-chlorophenyl)methanamine (0.867 mmol, 3 eq.), and *N,N-*diisopropylethylamine (1.73 mmol, 0.302 ml, 6 eq.) was heated to 140°C under microwave irradiation for 10 h. The reaction mixture was then concentrated under reduced pressure, and the residue purified by column chromatography on silica gel (Biotage SNAP 10 g column, 0-40% MeOH/EtOAc as the eluent, 28 CV) to afford the desired product in 59% yield (71 mg). LRMS (M + H)^+^: 407.4. Purity: 98% (UV/254 nm).

### Preparation of HP11 ((2*R*,3*R*,4*S*,5*R*)-2-(6-amino-8-((4-methoxybenzyl)amino)-9*H*-purin-9-yl)-5-(hydroxymethyl)tetrahydrofuran-3,4-diol)

HP11 was synthesized according to the synthesis procedure above from a mixture of 8-bromoadenosine (100 mg, 0.289 mmol, 1 eq.), (4-methoxyphenyl)methanamine (0.867 mmol, 3 eq.), and *N,N-*diisopropylethylamine (1.73 mmol, 0.302 ml, 6 eq.) was heated to 140°C under microwave irradiation for 10 h. The reaction mixture was then concentrated under reduced pressure, and the residue purified by column chromatography on silica gel (Biotage SNAP 10 g column, 0-40% MeOH/EtOAc as the eluent, 28 CV) to afford the desired product in 67% yield (78 mg). LRMS (M + H)^+^: 403.5. Purity: 100% (UV/254 nm).

### Preparation of HP12 ((2*R*,3*R*,4*S*,5*R*)-2-(6-amino-8-((naphthalen-1-ylmethyl)amino)-9*H*-purin-9-yl)-5-(hydroxymethyl)tetrahydrofuran-3,4-diol)

HP12 was synthesized according to the synthesis procedure above from a mixture of 8-bromoadenosine (100 mg, 0.289 mmol, 1 eq.), naphthalen-1-ylmethanamine (0.867 mmol, 3 eq.), and *N,N-*diisopropylethylamine (1.73 mmol, 0.302 ml, 6 eq.) was heated to 140°C under microwave irradiation for 10 h. The reaction mixture was then concentrated under reduced pressure, and the residue purified by column chromatography on silica gel (Biotage SNAP 10 g column, 0-40% MeOH/EtOAc as the eluent, 28 CV) to afford the desired product in 61% yield (74 mg). LRMS (M + H)^+^: 423.4. Purity: 96% (UV/254 nm).

## Results and discussion

### Crystal-based screening and identification of *pf*GRP78-NBD bound with eight nucleoside analogues

**FIGURE 1:**
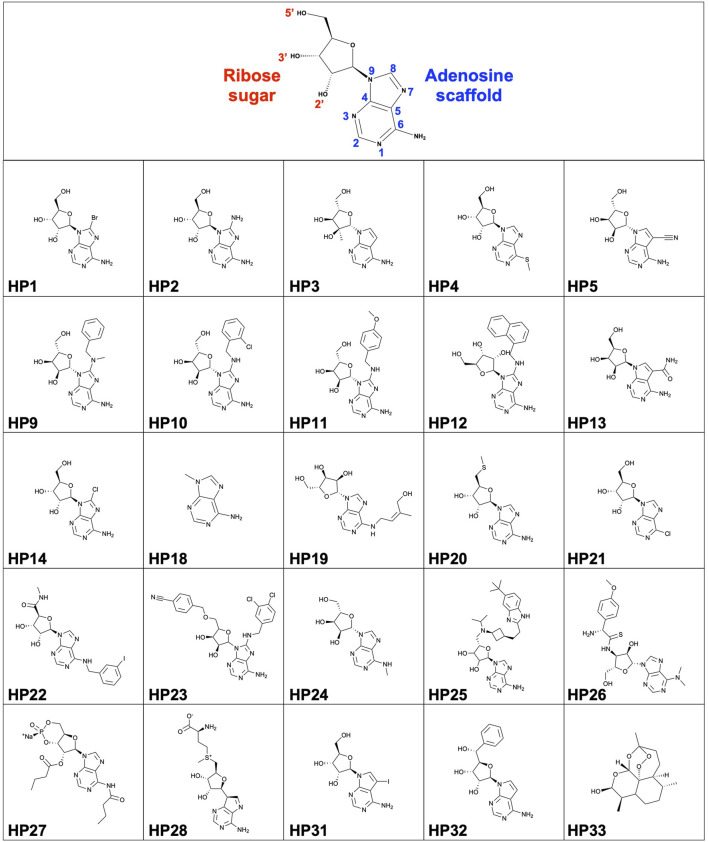
Structure of nucleoside scaffold and HP compound library (HP1-HP33).

**FIGURE 2:**
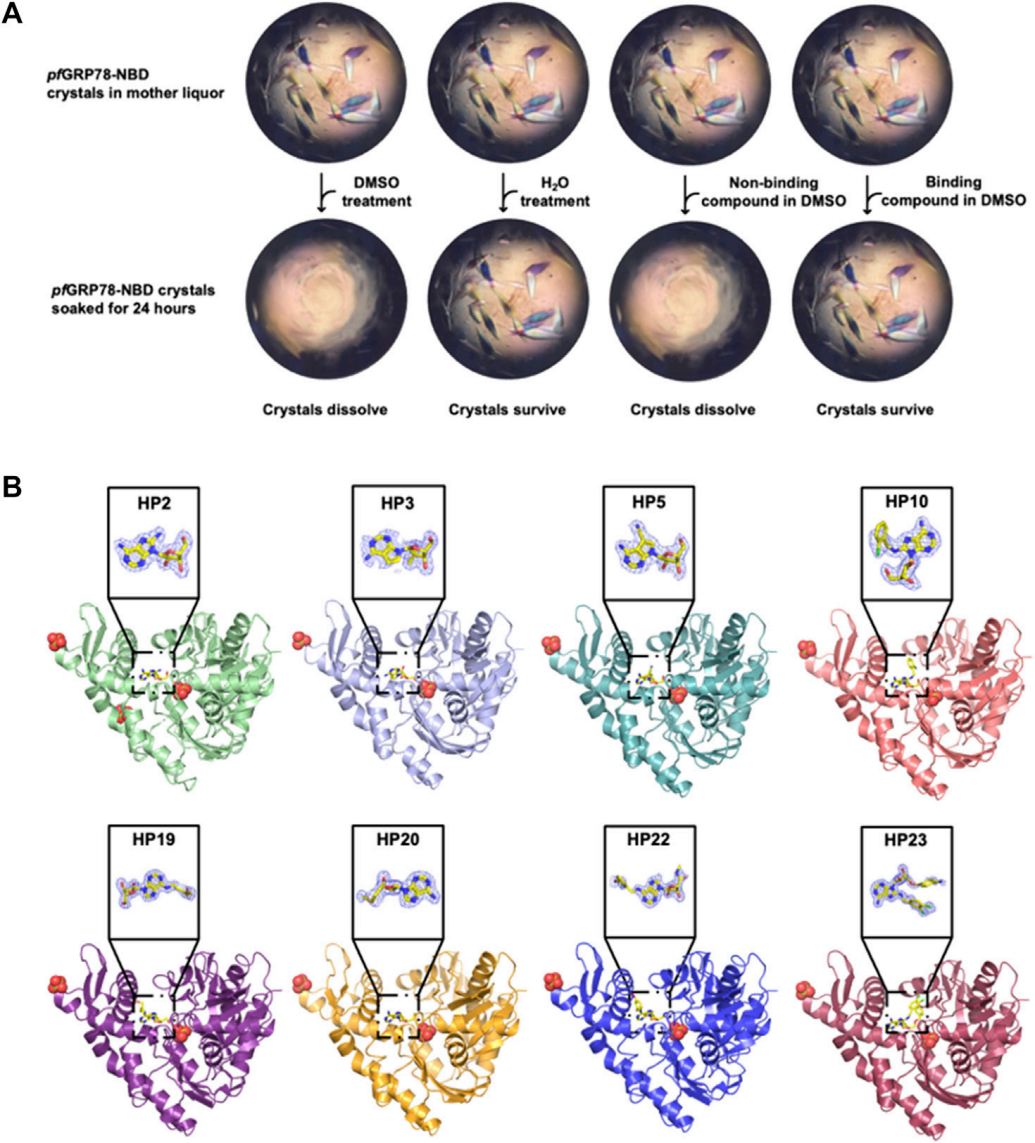
Crystal structures of *pf*GRP78-NBD bound with eight nucleoside analogues. **(A)** DMSO-driven Crystal-ligand replacement assay schematic. **(B)** Protein-ligand complex structures of *pf*GRP78-NBD with nucleoside analogues (HP2, HP3, HP5, HP10, HP19, HP20, HP22 and HP23) and corresponding electron density maps contoured to 2*F*_*o*_ *-F*_*c*_ at the 1 σ level showing molecular replacement of 8-bromoadenosine at the active site of *pf*GRP78-NBD.

In order to structurally characterize nucleoside analogue as potential anti-*plasmodium* chemical scaffolds in complexes with *pf*GRP78-NBD, we crystallized *pf*GRP78-NBD in complex with HP1 (8-bromoadenosine). We then soaked crystals with nucleoside analogues (Figure 1) using our novel DMSO-driven crystal-ligand replacement screening method. Certain compounds stabilized proteins in the crystal lattice, leading to the survival of the soaked crystals, resulting in the identification of protein-binding compounds later confirmed by x-ray crystallography. On the other hand, crystals soaked with non-binding compounds were dissolved within 24 h (Figure 2A). These results demonstrate our novel crystal-ligand DMSO soaking assay reduces the total number of crystal samples sent out for data collection and can be used for potential drug discovery efforts at large. Synchrotron beam time is a rate-limiting step in compound screening by conventional soaking approaches, thus the reduced number of samples sent out for data collection leads to a reduction in false-positives and expedited data analysis, representing a significant improvement in throughput. Using this approach, we successfully identified eight *pf*GRP78-NBD inhibitor complex structures to high resolutions. PDB entries for these *pf*GRP78-NBD nucleoside analogue complex structures are summarized in Table 1. These hit nucleoside analogs are HP2: 8-Aminoadenosine, HP3: 7-Deaza-2′-C-methyladenosine, HP5: Toyocamycin, HP10: (2R,3R,4S,5R)-2-(6-amino-8-((2-chlorobenzyl)amino)-9H-purin-9-yl)-5-(hydroxymethyl)tetrahydrofuran-3,4-diol), HP19: Trans-Zeatin Riboside, HP20: 5′-Methylthioadenosine, HP22: Piclidenoson and HP23: VER155008. In the active sites of all *pf*GRP78-NBD and nucleoside analogue complexes, electron density is well shaped for unambiguous modeling of eight hit compounds that replaced the 8-bromoadenosine compound (Figure 2B).

**TABLE 1:** X-ray data collection, refinement and validation statistics.

**Ligand**	**HP2**	**HP3**	**HP5**	**HP10**	**HP19**	**HP20**	**HP22**	**HP23**
**PDB ID**	8DIQ	8DIP	8DIS	8D1W	8DIY	8D20	8D22	8D24
**Beamline**	CLS 08B1-1	APS 19-ID	APS 19-ID	APS 19-ID	CLS 08B1-1	CLS 08B1-1	CLS 08B1-1	APS 23-ID-D
**Data Collection**								
Wavelength (Å)	1.5212	0.97918	0.97918	0.97918	1.5212	1.5212	1.5212	1.03322
Resolution (Å)	50.0–2.15	50.0–1.88	50.0–2.25	50.0–2.10	50.0–1.90	50.0–1.95	50.0–2.00	50.0–1.75
Space Group	P6_5_22	P6_5_22	P6_5_22	P6_5_22	P6_5_22	P6_5_22	P6_5_22	P6_5_22
No. of molecules in ASU	1	1	1	1	1	1	1	1
Unit cell parameters (Å)	a = 85.11, c = 292.07	a = 84.26, c = 291.78	a = 84.38, c = 292.49	a = 84.29, c = 292.22	a = 83.88, c = 291.23	a = 84.32, c = 291.75	a = 84.28, c = 292.20	a = 84.00, c = 292.60
No. of unique reflections^a^	34,525 (5360)	51,027 (8063)	30,364 (4774)	36,996 (5825)	49,029 (7724)	44,948 (6955)	42,527 (6624)	62,702 (9890)
Wilson B-Factor (Å^2^)	40.5	33.4	34.5	35.1	28.3	27.6	33.1	29.4
Redundancy^a^	19.1 (19.6)	18.9 (19.6)	18.9 (19.2)	18.9 (19.8)	18.5 (17.4)	19.2 (19.6)	18.7 (18.9)	19.0 (19.6)
R_sym_ (%)^a^ ^,^ ^b^	8.1 (60.9)	6.2 (46.4)	11.2 (52.8)	9.3 (49.5)	8.6 (51.3)	8.2 (50.7)	7.5 (50.4)	6.4 (51.2)
Average I/σ^a^	27.30 (5.55)	35.23 (6.36)	27.10 (8.99)	28.90 (8.26)	26.44 (5.67)	37.96 (9.25)	30.55 (6.13)	32.2 (6.01)
**Refinement**								
Resolution (Å)	48.73–2.15	45.62–1.88	48.80–2.25	45.66–2.10	48.58–1.90	45.63–1.95	48.75–2.00	48.81–1.75
Completeness (%)	97.8	99.9	99.9	99.9	99.9	97.3	99.2	99.9
R_work_/R_free_ (%)^c^	20.1/24.0	18.9/21.6	18.3/22.6	19.1/22.1	19.3/22.2	19.6/22.7	19.9/23.1	20.1/21.7
No. of atoms								
Protein	3003	3069	2987	2973	3005	3010	2982	3076
Ligand/ion	34	49	30	37	29	29	38	47
Water	239	277	236	265	310	321	302	301
RMSD bond length (Å)	0.011	0.013	0.012	0.012	0.014	0.013	0.014	0.013
RMSD bond angle (°)	1.712	1.781	1.729	1.780	1.816	1.800	1.835	1.860
Ramachandran Analysis								
Favored (%)	96.1	97.3	96.9	97.3	96.9	97.5	96.1	96.7
Outliers (%)	0.8	0.3	0.3	0.6	0.3	0.3	0.6	0.0

a Values in parentheses denote the highest-resolution shell.

b R_sym_ = Σ[(I−< I >)]/Σ(I), where I is the observed intensity and < I > is the average intensity.

c R_work_ = Σ[|F_obs_|−|F_calc_|]/Σ|F_obs_|, where |F_obs_| and |F_calc_| are magnitudes of observed and calculated structure factors. R_free_ was calculated as R_work_ using 5.0% of the data, which was set aside for an unbiased test of the progress of refinement.

### X-ray crystallographic analysis of *pf*GRP78-NBD: Ligand interactions at the ATP binding site

To identify specific modifications of adenosine advantageous for designing plasmodium specific compounds, we analyzed the complex structures of *pf*GRP78-NBD with the eight hit compounds identified through the crystal-ligand replacement screening method and compared the interactions to those present in hGRP78-NBD. Here we discuss the relevance of chemical modifications of the eight hit nucleoside analogues at the C8, C6, N7, 2′ and 5’ positions.

**FIGURE 3:**
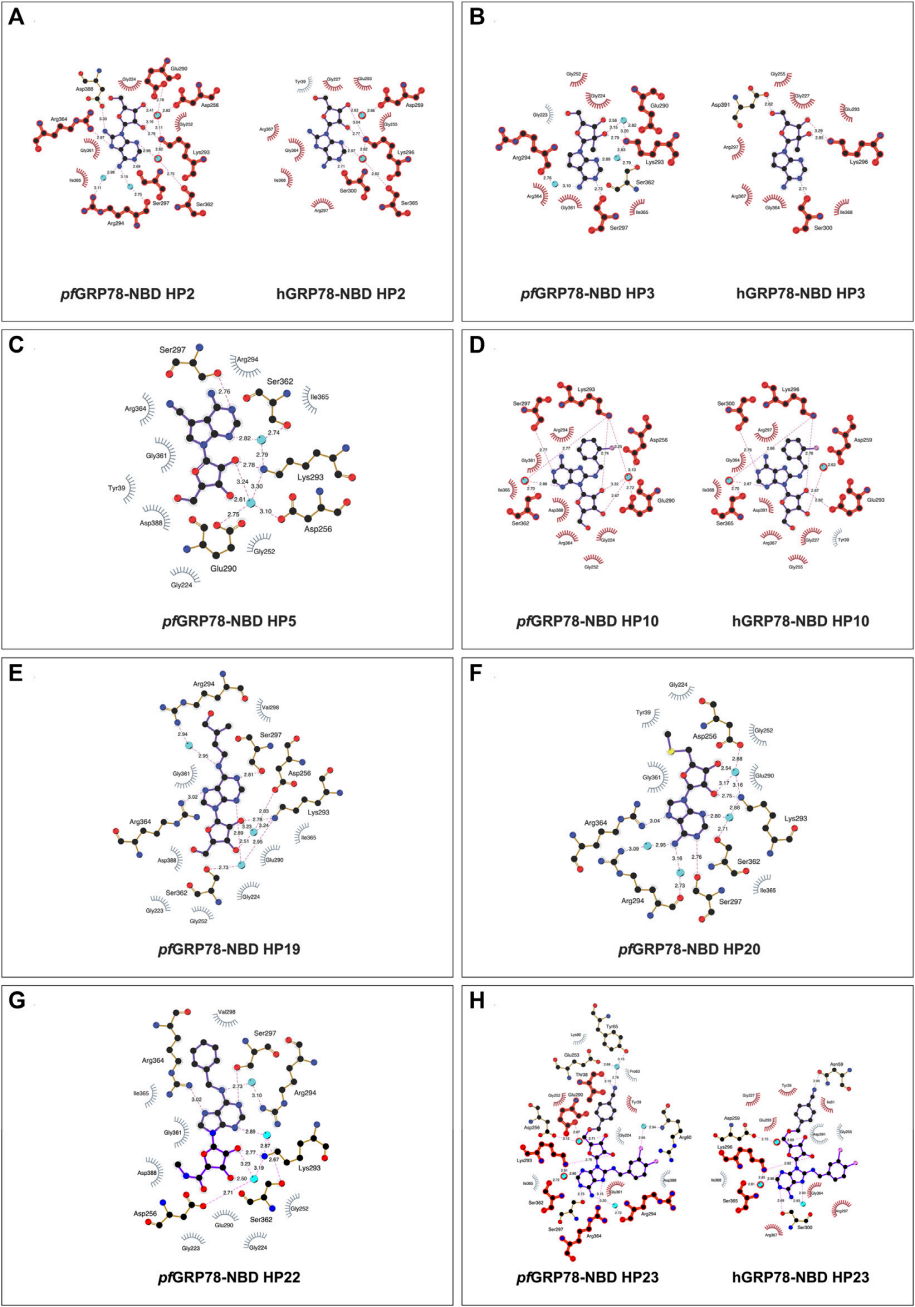
2D representation of protein-ligand interactions of hit nucleoside analogues identified from crystal-ligand soaking. **(A)** HP2, **(B)** HP3, **(C)** HP5, **(D)** HP10, **(E)** HP19, **(F)** HP20, **(G)** HP22 and **(H)** HP23. Ligand bonds are shown in purple, non-ligand bonds are shown in brown. C, N, O, S and Cl atoms are represented in black, blue, red, yellow and pink, respectively. Water atoms are represented in turquoise. Hydrophobic interactions are represented by grey spokes radiating towards the contacting ligand atoms and hydrogen bonds are represented by pink dashed lines with bond-lengths in Å. Red highlight represents amino acid equivalence between *pf*GRP78-NBD and hGRP78-NBD structure.

In Figure 3A, HP2 (8-Aminoadenosine) forms several hydrogen bonds with *pf*GRP78-NBD active site residues. HP2 forms direct hydrogen bonds with Lys293, Ser297 and Arg364. Four water molecules provide water-mediated hydrogen bonding with Asp256, Glu290, Arg294 and Ser362, further stabilizing HP2 to *pf*GRP78-NBD. Hydrophobic contacts are present with Gly224, Gly252, Gly361 and Ile365. The amino substituent at the C8 position directly interacts with Asp388, forming a hydrogen bond and has minimal hydrophobic interactions with Arg294. In comparison, the HP2-bound structure of hGRP78-NBD shows the conservation of most residues involved in HP2 binding (e.g., *pf*Lys293 and hLys296, *pf*Gly252 and hGly255). Specifically, a hydrogen bond and hydrophobic interactions with equivalent residues of hGRP78-NBD (hAsp391 and hArg297, respectively) are conserved, although the hydrogen bond is water-mediated in hGRP78-NBD. This suggests that the C8 amino substituent may not enhance specificity for *pf*GRP78-NBD, despite the improved stabilization this modification provides.

As depicted in Figure 3B, HP3 (7-Deaza-2′-C-methyladenosine) was used to assess how chemical modification at the 2′ hydroxyl group of the ribose sugar and the deaza-modified adenine moiety (i.e., N7 to C5) affect protein-ligand interactions at the ATP binding site of *pf*GRP78-NBD. It is evident that HP3 interacts with *pf*GRP78-NBD, but lacks hydrogen bonding with Arg364 due to the deaza substitution. Similarly, the HP3-bound structure of hGRP78-NBD shows no hydrogen bonding between hArg367 and HP3 either. In other nucleoside analogue-bound *pf*GRP78-NBD structures, a hydrogen bond is clear present between N7 and Arg364 (Table 2). The 2′ methyl substituent directs the 2′ hydroxyl group to form a hydrogen bond with Lys293, but it itself is involved in hydrophobic interactions with Glu290. In the HP3-bound structure of hGRP78-NBD, the N7 deaza substituent of HP3 is not involved in hydrogen binding, similar to that of *pf*GRP78-NBD. In addition, the 2′ methyl substituent of HP3 helps orient the 2′ hydroxyl group to form a hydrogen bond with hLys296 and has hydrophobic interactions with hGlu293 conserved in *pf*GRP78-NBD. The finding that the hydrogen bonding and hydrophobic contacts are conserved and indistinguishable between the *P*. *falciparum* and the human orthologue suggest that neither the deaza modification at the N7 position nor the 2′ methylation enhance selectivity for *pf*GRP78-NBD.

**TABLE 2:** *pf*GRP78-NBD & hGRP78-NBD Ligand Interaction Profile with Hit Nucleoside Analogues.

Compound	Hydrogen bond forming residues		Hydrophobic interaction forming residues	
	*pf*GRP78-NBD	hGRP78-NBD	*pf*GRP78-NBD	hGRP78-NBD
HP2	Asp256, Glu290, Lys293, Arg294, Ser297, Ser362, Arg364, Asp388	Asp259, Lys296, Ser300, Ser365	Gly224, Gly252, Gly361, Ile365	Tyr39, Gly227, Gly255, Glu293, Arg297, Gly364, Arg367, Ile368
HP3	Glu290, Lys293, Arg294, Ser297, Ser362	Lys296, Ser300, Asp391	Gly223, Gly224, Gly252, Gly361, Arg364, Ile365	Gly227, Gly255, Glu293, Arg297, Gly364, Arg367, Ile368
HP5	Asp256, Glu290, Lys293, Ser297, Ser362	—	Tyr39, Gly224, Gly252, Arg294, Gly361, Arg364, Ile365, Asp388	—
HP10	Asp256, Glu290, Lys293, Ser297, Ser362	Asp259, Glu293, Lys296, Ser300, Ser365	Gly224, Gly252, Arg294, Gly361, Arg364, Ile365, Asp388	Tyr39, Gly227, Gly255, Arg297, Gly364, Arg367, Ile368, Asp391
HP19	Asp256, Lys293, Arg294, Ser297, Ser362, Arg364	—	Gly223, Gly224, Gly252, Glu290, Val298, Gly361, Ile365, Asp388	—
HP20	Asp256, Lys293, Arg294, Ser297, Ser362, Arg364	—	Tyr39, Gly224, Gly252, Glu290, Gly361, Ile365	—
HP22	Asp256, Lys293, Arg294, Ser297, Ser362, Arg364	—	Gly223, Gly224, Gly252, Glu290, Val298, Gly361, Ile365, Asp388	—
HP23	Thr38, Arg60, Tyr65, Glu253, Asp256, Glu290, Lys293, Arg294, Ser297, Ser362, Arg364	Asn59, Asp259, Lys296, Ser300, Ser365	Tyr39, Pro63, Lys80, Gly224, Gly252, Gly361, Ile365, Asp388	Tyr39, Ile61, Gly227, Gly255, Glu293, Arg297, Gly364, Arg367, Ile368, Asp391

The active site architecture of *pf*GRP78-NBD with HP5 (Toyocamycin) is shown in Figure 3C. The HP5 compound also has deaza modification at the N7 position, which becomes C5. In addition, a cyano substituent is attached at the C5 position. Direct hydrogen bonds are made between Lys293 and the 2’ hydroxyl on the ribose moiety as well as Ser297 and the N1 position on the adenine moiety. Two water molecules provide water-mediated hydrogen bonds with Asp256, Glu290 and Ser362. Eight hydrophobic interactions are observed with Tyr39, Gly224, Gly252, Arg294, Gly361, Arg364, Ile365 and Asp388. The cyano substituent at the C5 position does not form direct hydrogen bonding with *pf*GRP78-NBD, but forms hydrophobic interactions with the aliphatic side chain of Arg294. Direct structural comparison with hGRP78-NBD cannot be made at this time due to the lack of structural data for the human orthologue in complex with HP5. However, most of the residues of *pf*GRP78-NBD interacting with HP5 are conserved in hGRP78-NBD, (e.g., hArg297, hSer300, hLys296) suggesting a similar binding mode for HP5, which involves no direct hydrogen bonding of the cyano substituent of HP5 with hGRP78-NBD.

The structure of HP10 ((2R,3R,4S,5R)-2-(6-amino-8-((2-chlorobenzyl)amino)-9H-purin-9-yl)-5-(hydroxymethyl)tetrahydrofuran-3,4-diol) in complex with *pf*GRP78-NBD is shown in Figure 3D. This compound has a modification at the C8 position, by adding an amino group followed by a chlorobenzyl moiety. Direct hydrogen bonding is observed with Lys293 and Ser297. Water-mediated hydrogen bonding is shown with Asp256, Glu290 and Ser362. Hydrophobic interactions are found with Gly224, Gly252, Arg294, Gly361, Arg364, Ile365 and Asp388. The C8 substituent shows minimal hydrophobic interactions with the hydrocarbon side chain of Arg294, although multiple hydrophobic interactions at the C8 substituent are observed in hGRP78-NBD involving Glu293, Lys296 and Ser300 (Figure 3D). This loose hydrophobic interactions at the active site of *pf*GRP78-NBD can be tightened by additional chemical modifications at this position to further occupy the open active site pocket, allowing for the development of more potent inhibitors specific to the *P. falciparum* protein.

Interactions of HP19 (Trans-Zeatin Riboside) with *pf*GRP78-NBD is shown in Figure 3E. This compound has a methylbutenol substituent at the C6 position. Direct hydrogen bonding of HP19 occurs with Lys293, Ser297 and Arg364. Three water molecules provide indirect hydrogen bonding with Asp256, Ser362 and Arg294. Hydrophobic interactions are also shown with Gly223, Gly224, Gly252, Glu290, Val298, Gly361, Ile365 and Asp388. The C6 substituent does not have any direct hydrogen bonding interactions with *pf*GRP78-NBD, although there is a water-mediated hydrogen bond that may provide additional stability. The C6 substituent forms hydrophobic interactions with Arg294 and Val298. Although the HP19-bound structure of hGRP78-NBD is not available, many active site residues of *pf*GRP78-NBD involved in HP19 interactions are conserved in hGRP78-NBD (e.g., hArg297, hArg367, hSer365). Interestingly, the C6 substituent forms hydrophobic interactions with *pf*Val298, which may not occur in hGRP78-NBD since *pf*Val298 is not conserved in hGRP78-NBD (hGln301). This difference can therefore be further probed for the development of compounds specific to *pf*GRP78-NBD.

Figure 3F shows the interactions between HP20 (5′-Methylthioadenosine) and *pf*GRP78-NBD. This compound has a modification at the 5′ position of the ribose sugar, with the deletion of the 5′ hydroxyl group and the addition of a methylated sulfur moiety. It is clear that no direct hydrogen bonding is observed at this modified 5′ position. Direct hydrogen bonding is however shown at other sites on HP20, which interact with Lys293, Ser297 and Arg364. Four water molecules provide water-mediated hydrogen bonding with Asp256, Arg294 and Ser362. There are six hydrophobic interactions shown with Try39, Gly224, Gly252, Glu290, Gly361 and Ile365. In *pf*GRP78-NBD, there are hydrophobic interactions of the 5′ substituent with Tyr39. Without the HP20-bound structure of hGRP78-NBD, we predict a similar binding mode of *P. falciparum* and human GRP78 for HP20 because of the conservation of HP20 binding residues between the two species including *pf*Tyr39 and hTyr39.

HP22 (Piclidenoson) interacts with the active site residues of *pf*GRP78-NBD in Figure 3G. This compound has modifications at both the 5′ position and the C6 position. Direct hydrogen bonds of HP22 are formed with Ser297, Lys293 and Arg364. Three water molecules provide water-mediated hydrogen bonds with Asp256, Arg294 and Ser362. Neither the 5′ modified group nor C6 modified group form direct hydrogen bonds with *pf*GRP78-NBD active site residues. However, there is a water-mediated hydrogen bond between the nitrogen atom of the C6 substituent and Arg294. There are eight hydrophobic interactions which include Gly223, Gly224, Gly252, Glu290, Val298, Gly361, Ile365 and Asp388. The 5′ modified substituent has hydrophobic interactions with Asp388 and Gly223 and the ring structure of the C6 substituent forms hydrophobic interactions with Val298. In the absence of the HP22-bound structure of hGRP78-NBD, we still can predict the binding mode of hGRP78-NBD for HP22. The 5’ substituent of HP22 can form hydrophobic interactions with hAsp391, equivalent to *pf*Asp388. However, the C6 substituent may not form hydrophobic interactions with the *pf*Val298-equivalent residue of hGRP78-NBD (hGln301). This suggests that further modification at the C6 position could be used in the development of the next generation of C6-modified nucleoside analogues that are more selective to *pf*GRP78-NBD.

The structure of pfGRP78-NBD with HP23 (VER155008) is shown in Figure 3H. This compound has modifications at the C8 and 5′ positions. Direct hydrogen bonding of HP23 is shown with Thr38, Lys293, Ser297 and Arg364. Five water molecules provide water-mediated hydrogen bonds with Tyr65, Arg60, Glu253, Asp256, Arg294, Ser362. Hydrophobic interactions are shown with Tyr39, Pro63, Lys80, Gly224, Gly252, Gly361, Asp388 and Ile365. Especially, the 5’ substituent makes direct interactions with Thr38 and water-mediated contacts with Glu253 and Tyr65. Moreover, a chlorine atom of the C8 substituent makes water-mediated hydrogen bonding with Arg60. In hGRP78-NBD, HP23 has direct hydrogen bonding with Asn59 and Lys296. Three water molecules mediate hydrogen bonds are present between N7 with Asp259, Lys296 and Ser365. When comparing *pf*GRP78-NBD and hGRP78-NBD, many interactions at the active site are conserved (e.g., *pf*Lys293 and hLys296, *pf*Ser362 to hSer365). These findings suggest HP23 binds to both *pf*GRP78 and hGRP78 indistinguishably. Different modification will be required to generate nucleoside analogues specific for *pf*GRP78-NBD.

### Identification of nucleoside analogues that thermally stabilize the *pf*GRP78-NBD

To further evaluate the interaction of pfGRP78-NBD with the hit nucleoside analogues, we performed thermal shift assays to measure the stability of protein-ligand complexes. Recombinant *pf*GRP78-NBD and hGRP78-NBD were used to evaluate potential preferential binding differences between the parasite versus host chaperone. We speculated that the limited structural variation between the highly conserved *P*. *falciparum* and human NBD of GRP78 may be an obstacle to developing selective antimalarial drugs, however discernable differences were noted between the thermal melting profiles. In this assay, the melting temperature (*T*_m_; temperature at which protein denaturation = 50%) is monitored by a fluorescent molecular probe binding to exposed hydrophobic regions on the protein and protein-ligand interactions are characterized by an observed shift in the melting temperature (Δ*T*_m_; difference in *T*_m_ between a sample treated with ligand minus the *T*_m_ of a sample in the absence of ligand). Thermal shifts values above 2°C were considered significant and are indicative of protein-compound interaction.

**FIGURE 4:**
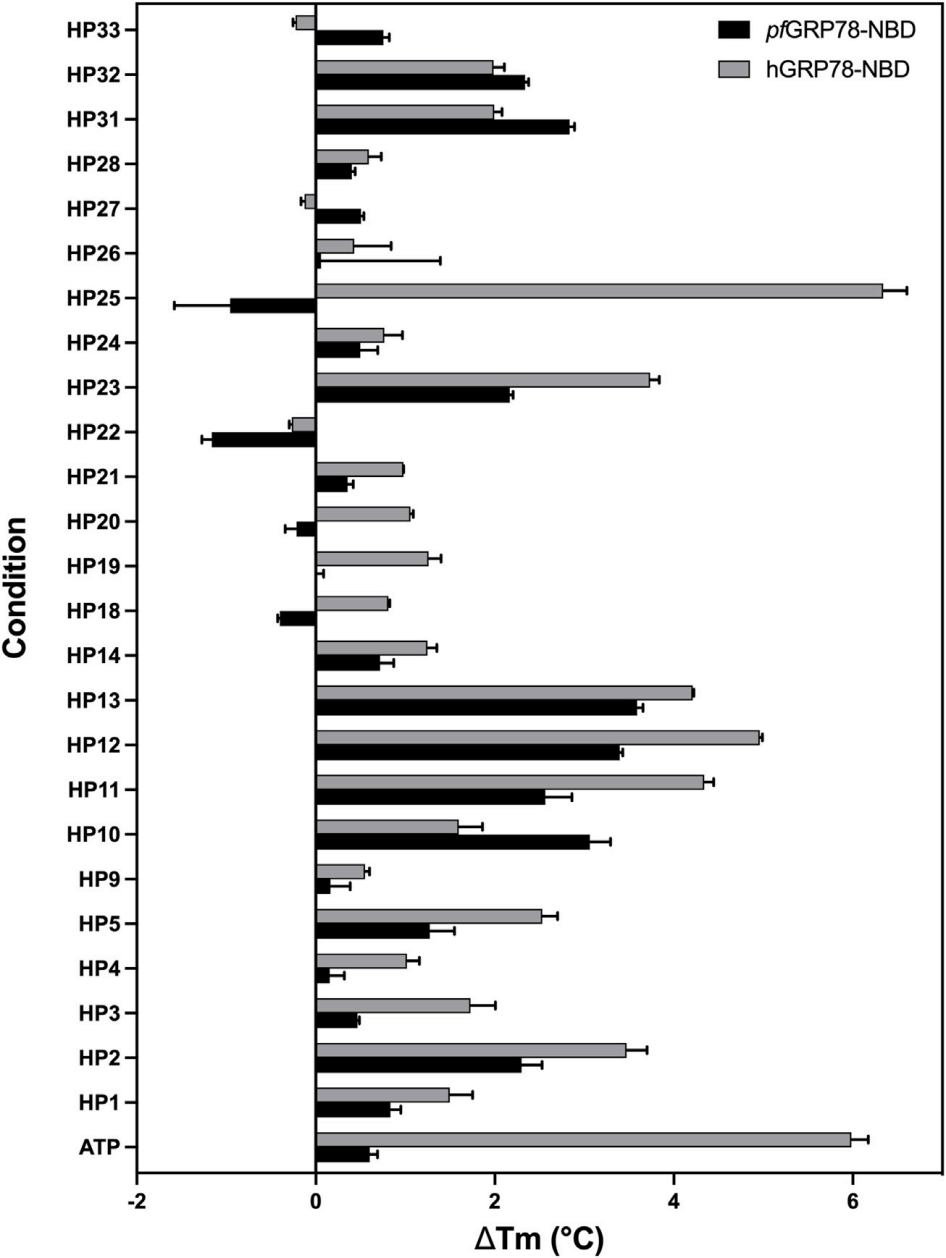
Interaction of nucleoside analogues on thermal stability of *pf*GRP78-NBD and hGRP78-NBD. Δ*T*_m_ is defined as the difference in *T*_m_ between protein with ligand minus the *T*_m_ of protein without ligand. Error bars represent standard error of the mean of four repeats.

The extent of thermal stabilization of *pf*GRP78-NBD and hGRP78-NBD in the presence of various nucleoside analogues to determine their binding to GRP78-NBDs (Figure 4). As a positive control, we used ATP as a model binding substrate. Both *pf*GRP78-NBD and hGRP78-NBD showed a positive thermal shift in the presence of ATP, compared to protein only. Although, the increase in melting temperature was more noticeable for hGRP78-NBD with a thermal shift of 5.6°C compared to only 0.5°C for *pf*GRP78-NBD. It is evident that numerous compounds interact with *pf*GRP78-NBD with different Δ*T*_m_ values, generating a unique chemical fingerprint. Eight out of the twenty-five screened compounds showed a significant effect on the *pf*GRP78-NBD thermal stability, these include: HP2, HP10, HP11, HP12, HP13, HP23, HP31 and HP32. Three of the eight compounds that showed a notable difference in the thermal stability were also identified using our DMSO crystal-ligand replacement screening, those being HP2, HP10 and HP23. Relative to the ATP control, the TSA data demonstrated stabilizing effects for many of the nucleoside analogues, with changes in melting temperature higher than ATP. These results are consistent with our hypothesis that the adenosine structural moiety common among compounds in this library would elevate the overall melting temperature and provides justification for further investigation.

In addition, screening the human homologue of GRP78-NBD generated different fingerprints indicative of differences in chemical sensitivity between the *P. falciparum* and human GRP78 chaperones that might be exploited in the design of selective inhibitors. Most notably, this can be seen in the case of HP25, which had a more noticeable (Δ*T*_m_ > 2 °C) shift for hGRP78, whereas the plasmodium chaperone was destabilized by HP25. These finding suggest the potential for designing inhibitors specific for *pfGRP78*. Interestingly, HP22 destabilized both *pf*GRP78-NBD and hGRP78-NBD in TSA, however our DMSO crystal-ligand replacement assay results clearly provide the crystal structure of *pf*GRP78-NBD with this nucleoside analogue bound at the active site. This result stresses the importance of performing orthogonal approaches to validate putative hit compounds.

## Conclusion

In summary, we purified recombinantly expressed *pf*GRP78-NBD and hGRP78-NBD and performed biophysical assays such as TSA to identify chemical moieties that could be used to generate more specific therapeutics to *P*. *falciparum*. We further characterized the active site of the NBD by providing new crystal structures of the *P. falciparum* GRP78 in complex with eight different nucleoside analogues using our innovative, high-throughput DMSO crystal-ligand replacement screening method. The results from the TSA and DMSO crystal-ligand replacement screening are in agreement and provide further validation of the hit compounds we identified in this study. Moreover, we provided proof of concept that protein crystals can tolerate DMSO soaking and stabilizing compounds remain intact, whereas non-binding compounds dissolve. Taken together, this study established an excellent platform for a rapid, stream-lined approach to compound screening, data collection and identification of protein-ligand complexes using x-ray crystallography. It is our belief that the development of our DMSO crystal-ligand screening approach can be applied in the larger context of drug discovery by minimizing time, cost and hours spent working to obtain protein-ligand co-crystal structures and will complement existing drug discovery approaches to identify and validate candidate drug targets. The research outcomes presented here will play a fundamental role in future studies to generate a refined adenosine chemotype containing structure-driven modifications on the adenosine scaffold specific and selective to *pf*GRP78.

## Data Availability

The datasets presented in this study can be found in online repositories. The names of the repository/repositories and accession number(s) can be found in the article/supplementary material.
